# Treatment of Otorrhœa

**Published:** 1893-06-17

**Authors:** 


### The Hospital Clinic.

[*The Editor will be glad to receive offers of co-operation and contributions from members of the profession. All letters should be addressed to* The Editor, The Lodge, Porchester Square, London, W.]

### NEWCASTLE-ON-TYNE THROAT AND EAR HOSPITAL.

### Treatment of Otorrhœa (Discharge from the Ear).

(*Continued from page 349, Vol. XIII.*)

When suppurative disease has existed for some considerable time—perhaps years—in the middle ear certain changes more or less serious take place. The inflammatory action attacking the mucous membrane lining the tympanic cavity may destroy this, and, penetrating into the internal ear, there cause destruction of the delicate structures comprising the membranous labyrinth. In this way the basilar membrane and Corti's organ may become diseased, and total deafness result. Such an occurrence is of special frequency when severe inflammation attacks the tympanic cavity in childhood, more especially during an attack of scarlet fever in a subject where adenoid growths are also present.

In very few cases of long standing suppurative disease of the tympanum do we find that the tympanic membrane and the ossicles have been able to withstand the ravages of the process. Their mucous membrane lining becomes first destroyed, after which the corroding action of the pus soon softens and dissolves away the bonelets of the ear. The first to suffer is, perhaps, the anvil bone. Next to follow is the malleus, or hammer bone. The stirrup bone—the one that communicates with the internal ear, or labyrinth—seems to have a more robust existence than the others, and is oftener found left behind, and if inflammatory action has not immovably fixed it in the oval window of the vestibule of the labyrinth, a fair amount of hearing remains, which may be greatly improved by the addition of an artificial drum after the suppuration of the tympanum has ceased.

In some cases the inflammatory action in the tympanum binds the parts together, so that all outlet for the pus formed is cut off. This damming up of pus in the cavity of the tympanum is a serious matter, inasmuch as the pus may be forced in other directions, more especially towards the brain, there to cause inflammation and abscess. To counteract such serious consequences the surgeon steps in, and by a suitable operation cuts out the drum head and ossicles. At the Newcastle-on-Tyne Throat and Ear Hospital the following procedures are adopted by Dr. Robertson: The tympanum is carefully disinfected by carbolic solution, then the parts are thoroughly soaked in a 20 per cent. solution of cocain, which, since the cavity of the tympanum is open, anæsthetises the parts fairly well. Seeing that the procedure is to effect drainage of the attic of the tympanum, and thus prevent pus escaping through the thin bony plate, which is all that separates the pus in the attic from the brain, the hammer bone is the part we endeavour to remove. The head of this bone reaches up into the attic, and consequently the space left by its removal allows free escape of any fluid lodged in the attic. To effect its removal a small knife is used to free it from the membrane on each side. Then a small rectangular knife is inserted inside the neck of the bone, and the tensor tympani tendon is cut. All this is done under cocain and a good light, the patient sitting up. These steps can be gone about leisurely, the cocain acting well not only as an anæsthetic, but also as a hæmostatic. Once the malleus is freed both from the remains of the tympanic membrane and the tensor tympani tendon, it is noticed to be freely moveable, in fact, just when the tensor tympani is cut it is noticed to spring outwards, showing that there had been previously considerable abnormal contraction of the parts acting injuriously on hearing, for as often also does the patient at once state that his hearing is better. As the next, the final removal of the bone is somewhat painful—more than the majority of patients will bear, but at the worst is of but short duration—advantage is taken of nitrous oxide gas as an anæsthetic, because it permits of the patient sitting up, and therefore favourable to better illumination of the parts being operated upon. When the patient goes under, the forceps already placed in position are made to grasp the body of the bone (hammer bone) as far as possible, and it is smartly extracted from its bed as quickly as possible. Any blood effused is mopped up, and with a bent probe or blunt hook search is made for the anvil, but not too far up into the attic in case injury to the covering to the attic might ensue, and then fatal damage to the brain occur. Often, indeed, from causes mentioned, this bone, the anvil bone, the most feebly nourished of the three small bones of the ear, is found wanting, from probably long before having been destroyed by disease.

The stronger vitality of the malleus is probably due to its insertion into the membrana tympani which supports it and conveys a large number of bloodvessels to its neighbourhood, The subsequent treatment of such a case is simplicity itself. Hæmorrhage is checked, the parts are lightly dusted with iodoform, and iodoform gauze is inserted to exercise gentle pressure and restrain the production of pus. Next morning the gauze dressing is removed and the tympanum swabbed out with a pledget of cotton wool dipped in hydrogen peroxide or some such disinfecting agent, and the iodoform gauze dressing renewed. The experience hitherto gained of this operation is as regards cessation of the formation of pus, highly favourable provided that the antrum of the mastoid is not involved in the suppurative process. The other results that accrue are of importance. With the subsidence of the inflammatory process in the tympanum its mucous membrane looses its thick and pulpy consistence. If the stirrup bone is present and movable in the oval window and the round window not obstructed then hearing is often greatly benefited. An even greater benefit still is gained if after all diseased processes in the tympanum have subsided an artificial drum is accurately placed in the cavity. This tends to shore up the vibrations of sound on to the two windows mentioned, and by so much increases their intensity. In this way useful hearing is oftener attained over and above the enhanced security of life secured through the cure of the inflammatory condition in the ear which gave rise to the discharge.

In the event of the inflammatory condition having proceeded to and deeply affected the mucous membrane of the antrum of the mastoid process, then a different surgical procedure must be had recourse to. A new and comparatively safe method of operating in such cases will be described at a future date. With regard to this antral cavity in the mastoid, it may be remarked that it is lined by a mucous membrane directly continuous with the cavity of the tympanum, but separated from it by a somewhat restricted neck. Now this narrow part often becomes further narrowed by swelling of its lining membrane, so that in inflammation in the antrum fluid collects there, and being unable to drain off into the tympanum it is confined in the antrum. A stage arrives when it must find some way of escape, and this it does often in the direction of the brain, with disastrous consequences, unless released by timely operation.

